# Supplementary material for: End Sequence Analysis Toolkit (ESAT) expands the extractable information from single-cell RNA-seq data
Source: Genome Res. 2016 Oct;26(10):1397–410. doi: 10.1101/gr.207902.116 (PMC5052061; doi:10.1101/gr.207902.116)
Supplement: Supplemental Material [file supp_gr.207902.116_Supp_Materials.tar.gz › ESAT-esat_v0.1/doc/html/classbroad_1_1pda_1_1geneexpression_1_1agilent_1_1_agilent_array_stats.html]

ESAT: broad.pda.geneexpression.agilent.AgilentArrayStats Class Reference


ESAT


- Main Page
- Related Pages
- Packages
- Classes
- Files

- Class List
- Class Index
- Class Hierarchy
- Class Members

All Classes Namespaces Files Functions Variables Enumerator Pages

- broad
- pda
- geneexpression
- agilent
- AgilentArrayStats

Public Member Functions |
List of all members

broad.pda.geneexpression.agilent.AgilentArrayStats Class Reference

|  |  |
| --- | --- |
| Public Member Functions | |
|  | AgilentArrayStats (String line, String headerLine) |
|  | |
| double | getNonControl1PercentileSignal () |
|  | |
| double | getNonControl50PercentileSignal () |
|  | |
| double | getNonControl99PercentileSignal () |
|  | |
| int | getNumberOfBackgroundNonUniformFeaturesOverlimit () |
|  | |
| int | getNumberOfBackgroundPopulationOverlimit () |
|  | |
| int | getNumberOfFoundFeatures () |
|  | |
| int | getNumberOfNonUniformFeaturesOverlimit () |
|  | |
| int | getNumberOfPopulationOverlimit () |
|  | |
| int | getNumberOfSaturatedProbes () |
|  | |
| double | getSaturationSignalValue () |
|  | |
| double | getSpikeInDetectionLimit () |
|  | |
| int | getTotalNumberOffeatures () |
|  | |
| double | getPercentFeaturesWellAboveBackground () |
|  | |
| double | getNormalizationFactor () |
|  | |

## Constructor & Destructor Documentation

|  |  |  |  |
| --- | --- | --- | --- |
| broad.pda.geneexpression.agilent.AgilentArrayStats.AgilentArrayStats | ( | String | *line*, |
|  |  | String | *headerLine* |
|  | ) |  |  |

## Member Function Documentation

|  |  |  |  |  |
| --- | --- | --- | --- | --- |
| double broad.pda.geneexpression.agilent.AgilentArrayStats.getNonControl1PercentileSignal | ( |  | ) |  |

|  |  |  |  |  |
| --- | --- | --- | --- | --- |
| double broad.pda.geneexpression.agilent.AgilentArrayStats.getNonControl50PercentileSignal | ( |  | ) |  |

|  |  |  |  |  |
| --- | --- | --- | --- | --- |
| double broad.pda.geneexpression.agilent.AgilentArrayStats.getNonControl99PercentileSignal | ( |  | ) |  |

Here is the caller graph for this function:

|  |  |  |  |  |
| --- | --- | --- | --- | --- |
| double broad.pda.geneexpression.agilent.AgilentArrayStats.getNormalizationFactor | ( |  | ) |  |

|  |  |  |  |  |
| --- | --- | --- | --- | --- |
| int broad.pda.geneexpression.agilent.AgilentArrayStats.getNumberOfBackgroundNonUniformFeaturesOverlimit | ( |  | ) |  |

|  |  |  |  |  |
| --- | --- | --- | --- | --- |
| int broad.pda.geneexpression.agilent.AgilentArrayStats.getNumberOfBackgroundPopulationOverlimit | ( |  | ) |  |

|  |  |  |  |  |
| --- | --- | --- | --- | --- |
| int broad.pda.geneexpression.agilent.AgilentArrayStats.getNumberOfFoundFeatures | ( |  | ) |  |

|  |  |  |  |  |
| --- | --- | --- | --- | --- |
| int broad.pda.geneexpression.agilent.AgilentArrayStats.getNumberOfNonUniformFeaturesOverlimit | ( |  | ) |  |

|  |  |  |  |  |
| --- | --- | --- | --- | --- |
| int broad.pda.geneexpression.agilent.AgilentArrayStats.getNumberOfPopulationOverlimit | ( |  | ) |  |

|  |  |  |  |  |
| --- | --- | --- | --- | --- |
| int broad.pda.geneexpression.agilent.AgilentArrayStats.getNumberOfSaturatedProbes | ( |  | ) |  |

|  |  |  |  |  |
| --- | --- | --- | --- | --- |
| double broad.pda.geneexpression.agilent.AgilentArrayStats.getPercentFeaturesWellAboveBackground | ( |  | ) |  |

|  |  |  |  |  |
| --- | --- | --- | --- | --- |
| double broad.pda.geneexpression.agilent.AgilentArrayStats.getSaturationSignalValue | ( |  | ) |  |

|  |  |  |  |  |
| --- | --- | --- | --- | --- |
| double broad.pda.geneexpression.agilent.AgilentArrayStats.getSpikeInDetectionLimit | ( |  | ) |  |

Here is the caller graph for this function:

|  |  |  |  |  |
| --- | --- | --- | --- | --- |
| int broad.pda.geneexpression.agilent.AgilentArrayStats.getTotalNumberOffeatures | ( |  | ) |  |

---

The documentation for this class was generated from the following file:

- java/broad/pda/geneexpression/agilent/AgilentArrayStats.java


---

Generated on Fri Aug 15 2014 15:11:00 for ESAT by  

 1.8.7
